# Re-Examination Characterization and Screening of Stripe Rust Resistance Gene of Wheat *TaPR1* Gene Family Based on the Transcriptome in Xinchun 32

**DOI:** 10.3390/ijms26020640

**Published:** 2025-01-14

**Authors:** Tingting Sun, Niannian Yan, Qi Liu, Tingyu Bai, Haifeng Gao, Jing Chen

**Affiliations:** 1 Key Laboratory of the Pest Monitoring and Safety Control of Crops and Forests of the Xinjiang Uygur Autonomous Region, College of Agronomy, Xinjiang Agricultural University, Urumqi 830052, China; yep_ohh@163.com (T.S.); 17690712604@163.com (N.Y.); 18399059942@163.com (T.B.); chenj@xjau.edu.cn (J.C.); 2 Key Laboratory of Prevention and Control of Invasive Alien Species in Agriculture & Forestry of the North-Western Desert Oasis, Ministry of Agriculture and Rural Affairs, Urumqi 830052, China; 3 Institute of Plant Protection, Xinjiang Academy of Agricultural Sciences/Key Laboratory of Integrated Pest Management on Crop in Northwestern Oasis, Ministry of P.R. China, Urumqi 830091, China; xghf20044666@163.com

**Keywords:** wheat stripe rust, *Puccinia striiformis* f. sp. *tritici* (*Pst*), pathogenesis-related protein-1 (PR1), RNA sequencing, gene family analysis

## Abstract

Pathogenesis-related protein-1 (PR1) encodes a water-soluble protein produced in plants after pathogen infection or abiotic stimulation. It plays a crucial role in plant-induced resistance by attacking pathogens, degrading cell wall macromolecules and pathogen toxins, and inhibiting the binding of viral coat proteins to plant receptor molecules. Compared to model plants, the mechanism of action of PR1 in wheat remains underexplored. In this study, the recently published wheat genome database (IWGSC RefSeq V2.1) was used to identify 83 genes in the *TaPR1* gene family. Compared to previous work, the duplicate genes were removed and we corrected misannotated genes. Fourteen *TaPR1* genes involved in the wheat–*Pst* interaction were identified based on RNA sequencing from Xinchun 32. The expression patterns of eight genes were validated using qRT-PCR, and the results showed that PR1 was highly expressed following *Puccinia striiformis* f. sp. *tritici* (*Pst*) infection. This study enhances previous research on wheat PR1, contributing to a more comprehensive understanding of the *TaPR1* gene family and providing a reference for the screening of more broad-spectrum and high-resistance wheat populations.

## 1. Introduction

Wheat (*Triticum aestivum* L.) is one of the most important food crops in the world. However, its yield and quality are affected by different diseases; the most prevalent wheat disease is stripe rust, caused by *Puccinia striiformis* Westend. f. sp. *tritici* (*Pst*) [1]. An outbreak of wheat stripe rust can rapidly result in stunted growth, reduced grain numbers per spike, shriveled grains, and decreased grain weights [2]. In susceptible wheat varieties, the grain yield losses can reach up to 100% [3]. Currently, approximately 88% of the world’s wheat is susceptible to stripe rust, leading to estimated annual losses of 5.47 million tons of grain [4]. Numerous studies have demonstrated that breeding resistant varieties is the most economical and effective strategy to control this disease [5]. There are 86 officially designated anti-stripe rust genes worldwide (*Yr1-Yr86*) [6]. Identifying new sources of resistance, understanding their mechanisms, and developing new transgenic strategies to modify wheat varieties have become increasingly crucial in mitigating the damage caused by stripe rust.

Pathogenesis-related proteins (PRs) are a class of low-molecular-weight antimicrobial substances associated with plant defense mechanisms against pathogens. Currently, 17 PR families are known to be widely present in wheat, including thaumatin-like proteins, chitinases, peroxidases, glucanases, and proteases [7]. In the early 1970s, the PR family was first reported in tobacco plants infected by the tobacco mosaic virus [8]. Subsequently, PRs have been extensively identified in plants, such as *Arabidopsis*, tomato, and potato [7,9]. In general, the PR family encoded multiple proteins involved in pathogen defense through various mechanisms [10]. PR1 is a member of the PR family, possessing antifungal and antiviral functions that help plants to resist stress. It also indicates the acquisition of the disease-induced systemic acquisition resistance (SAR) defense signal [11]. Studies have shown that PR1 is a salicylic acid (SA) response gene and a major participant in the SA pathway [12], which can be induced by the application of salicylic acid in wheat [13]. However, the role of PR1 in wheat–*Pst* interaction has been little studied, and the molecular mechanism of PR1 still needs to be examined.

High-quality reference genome sequences and annotations for a variety of plants have been developed, such as wheat, and continuous updates have made them more detailed, providing resources for systematic studies of gene families like the PR family. In this study, the common wheat reference genome sequence (IWGSC RefSeq V2.1) was used to identify PR1 genes in wheat, rice, *Arabidopsis*, and tobacco, and we performed analyses on all *TaPR1* families, including chromosome localization, the conserved domains and motifs, and collinear analysis. Subsequently, we selected wheat Xinchun 32 for transcriptome sequencing analysis and analyzed the expression pattern of *TaPR1* in the transcriptome. In this study, the gene family analysis was conducted using the IWGSC RefSeq V2.1 data to obtain more comprehensive results, and 14 *TaPR1* genes that were upregulated in XC 32 after inoculation with *Pst* were identified. This work provides a valuable complement to previous research, with the aim of contributing to the enhancement of wheat’s resistance to stripe rust and accelerating the breeding of disease-resistant wheat varieties.

## 2. Results

### 2.1. PR1 Family Identification

The updated version of the wheat genome database IWGSC RefSeq V2.1 provides the more accurate and detailed characterization of the PR1 family members compared to the previous version [14]. A total of 83 non-redundant *TaPR1* genes were identified in IWGSC RefSeq V2.1 through BLASTP and the subsequent examination of the conserved domain database. Following the general nomenclature rules for wheat genes, we renamed these 83 *TaPR1* genes as *TaPR1-1* to *TaPR1-83*, as shown in Table S1. The details of the genes/proteins are listed in Table S2. The amino acid lengths of the *TaPR1* members ranged from 149 to 312 amino acids (aa), the theoretical isoelectric point (PI) values ranged from 4.23 to 11.68, and the molecular weights ranged from 16.88 kDa to 33.63 kDa.

**Figure 1:**
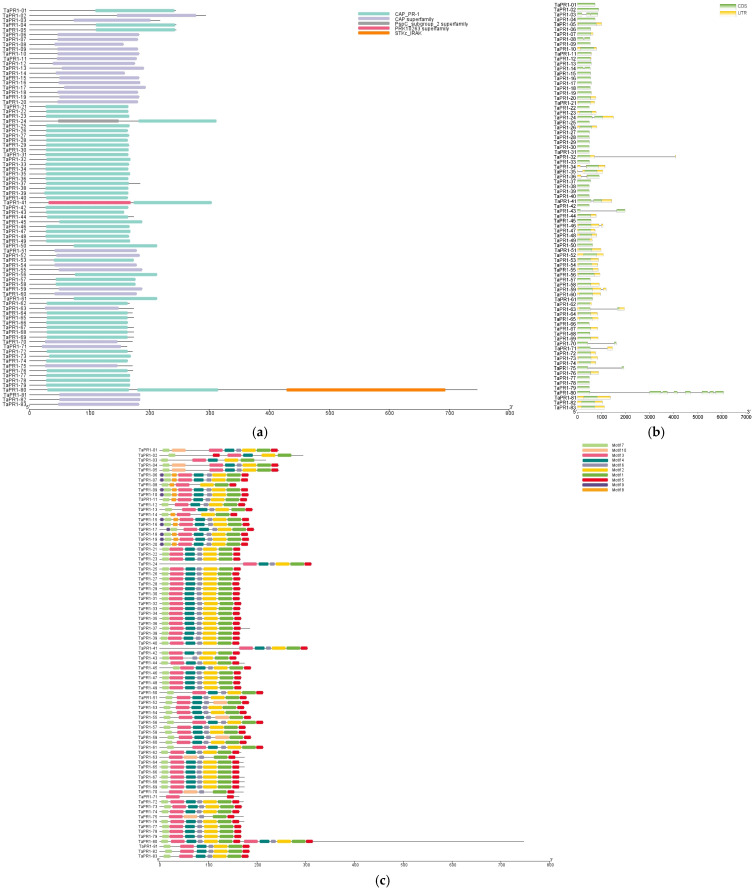
(**a**) Conserved domains; (**b**) gene structure; (**c**) conserved motifs of wheat *TaPR1* proteins. The image was created by TBtools.

Several published studies have utilized the old version (IWGSC RefSeq V1.1) of the wheat genome for the identification of *TaPR1* [15]. The findings of the current study were compared with those of these previous studies. Liu et al., 2023 identified 86 *TaPR1* genes in IWGSC RefSeq V1.1, all of which contained a cysteine-rich protein (CAP) domain, with some members containing either a PRK14950, PRK10263, or two STKt_IRAK domains. However, our study identified 83 *TaPR1* genes in IWGSC RefSeq V2.1, which included five domains, as shown in Figure 1a. Additionally, we identified the PspC_subgroup_2 superfamily domain, which differs from those reported in previous studies.

In addition, during the bidirectional comparison process, it was discovered that the gene *TraesCSU02G233000.1* identified in previous studies corresponded to *TraesCSU03G0404500.1* and *TraesCSU03G0309900.1*; *TraesCS5B02G443500.1* was identified as *TraesCS5B03G1089600.1* and *TraesCS5B03G1089800.1* in the IWGSC RefSeq V2.1 data. Similarly, *TraesCSU02G076600.1*, *TraesCSU02G2029001*, and *TraesCSU02G233000.1* were identified as *TraesCS5D03G0981300LC.1*, while *TraesCS5B02G443600.1* and *TraesCS5B02G443700.1* were identified as *TraesCS5B03G1089900LC.1*. Additionally, *TraesCS7D02G099600.1* and *TraesCSU02G095300.1* were matched to *TraesCS7D03G0226600.1*. *TraesCS3A02G525700.1* and *TraesCS5B02G443000.1* were identified as *TraesCS3A03G1240400.1*. Multiple sequence alignments suggested that these discrepancies may have arisen from missplicing or misannotation in the 2018 genome version. This indicates that further studies based on the IWGSC RefSeq V2.1 data are highly necessary.

Based on the IWGSC RefSeq V2.1 dataset, the duplicate genes caused by incorrect annotations and splicing errors were removed, leading to a more complete and accurate description of this important gene family. Additionally, intraspecies and interspecies collinearity analyses were conducted, aiding the study of the evolution of the PR1 family in wheat.

### 2.2. Phylogenetic Analysis of TaPR1 Genes

**Figure 2:**
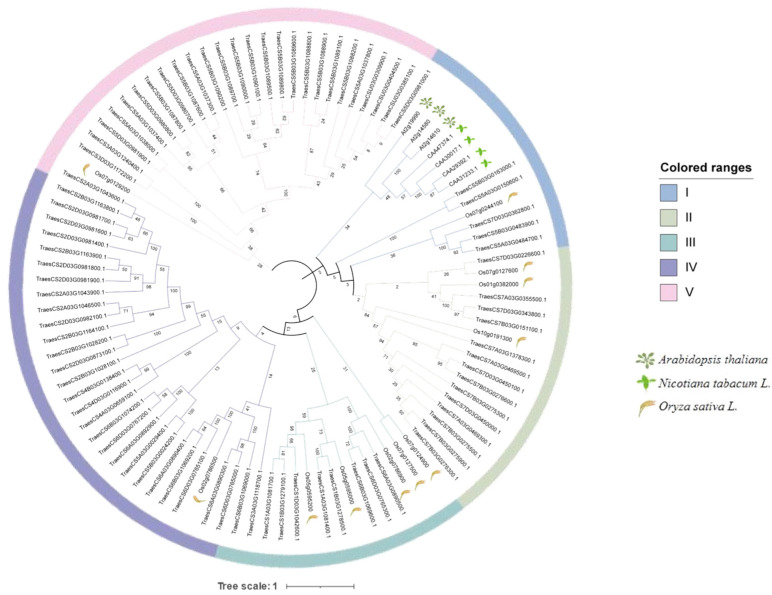
Phylogenetic tree of 101 PR1 proteins in four species (wheat, rice, tobacco, *Arabidopsis*). The image was created by MEGA X.

The results of the phylogenetic analysis of the PR1 family are shown in Figure 2. A total of 101 PR1 members were identified in four species: 83 in wheat, 11 in rice, 4 in tobacco, and 3 in *Arabidopsis*. All full-length amino acid sequences of the PR1 members were used to construct a phylogenetic tree to determine the evolutionary relationships of the *TaPR1* genes in wheat, rice, tobacco, and *Arabidopsis* and to further classify the PR1 members. Consequently, the PR1 members were divided into five groups based on the phylogenetic analysis. The *TaPR1* genes had corresponding sequences in each group, where rice was mainly classified into three groups: I, II, and III. Tobacco was mainly classified as group I, and *Arabidopsis* was classified as groups I and III. These results indicate that the *TaPR1* genes in wheat are more closely related to those in rice than to those in tobacco and *Arabidopsis*.

### 2.3. Structural Characteristics of the TaPR1 Gene Family

The characteristics of the *TaPR1* genes and proteins were described based on the conserved domain, conserved motif, and gene structure analysis, as shown in Figure 1; the domain prediction information is shown in Table S3. Five domains were identified, and the figure shows that each *TaPR1* gene contained the CAP domain, and three genes contained the STKt_IRAK, PRK10263 superfamily, and PspC_subgroup_2 superfamily. The MEME online software was used to identify conserved motifs, revealing 10 motifs in the *TaPR1* gene family. The results showed that all *TaPR1* genes contained motifs 1, 2, 3, 4, 5, 6, and 7. The gene structure analysis showed that most *TaPR1* genes were composed of CDS and UTR. Cis-acting elements are classified into three major types: the light response, hormone response, and stress response. The *TaPR1* gene contains ABA response elements, MeJA response elements, and SA response elements; the result is shown in Figure S1. These results indicate that most of the *TaPR1* gene families have the same gene structure and conserved domain, indicating that they are highly similar in function and sequence.

### 2.4. Chromosomal Distribution of TaPR1 Genes

**Figure 3:**
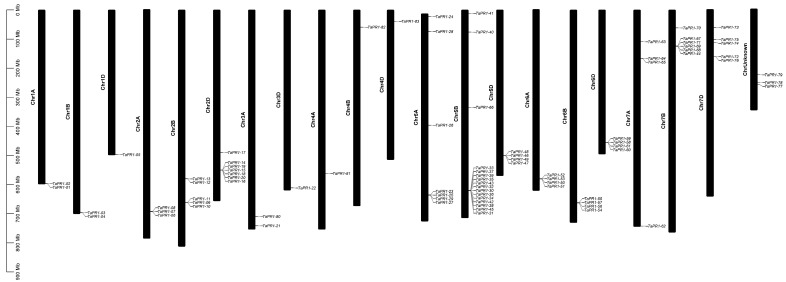
Chromosome distributions of *TaPR1* genes in wheat. The image was created by TBtools.

Based on the gene annotation information, 83 *TaPR1* genes were located on the IWGSC Refseq V2.1 chromosome. The 83 *TaPR1* genes were distributed throughout the whole genome. As shown in Figure 3, chromosome 5B contained the largest number of *TaPR1* genes (16), followed by chromosomes 2D and 5A, each with seven *TaPR1* genes; chromosomes 2B, 7B, and 7D with five, six, and five genes, respectively; chromosomes 5D, 6A, 6B, 6D, and 7A, each with four genes; chromosomes 1A, 1B, 2A, and 3A, with two, two, three, and two genes, respectively; and chromosomes 1D, 3D, 4A, 4B, and 4D contained only one gene. In addition, the three genes *TaPR1-77*, *TaPR1-78*, and *TaPR1-79* were located on unknown chromosomes. This phenomenon might have been caused by incorrect splicing or misannotation as the new and old versions were being updated.

### 2.5. Collinearity Analysis of TaPR1 Gene Family

**Figure 4:**
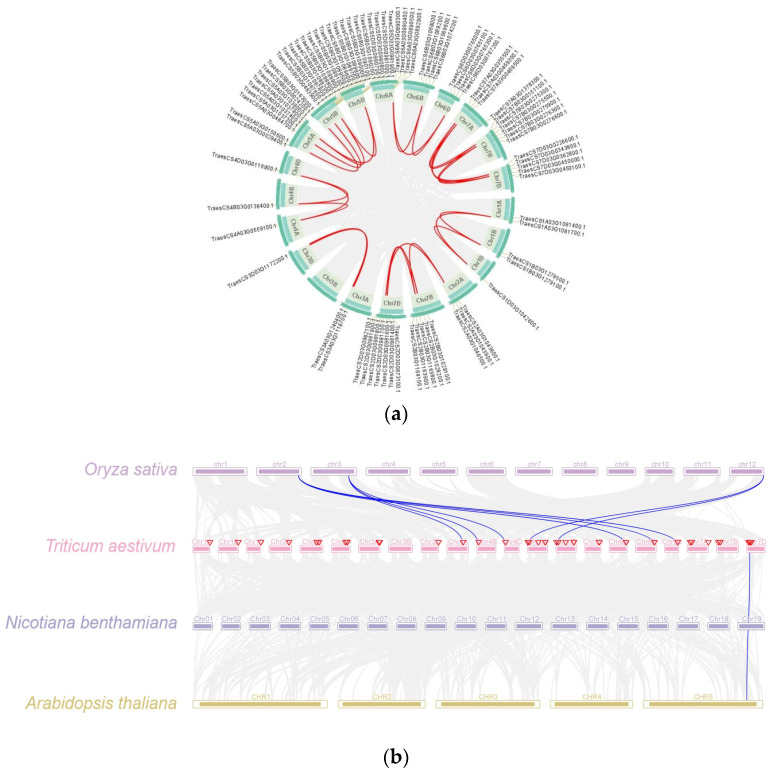
Collinearity analysis of the *TaPR1* gene family. (**a**) Intraspecies collinearity analysis of *TaPR1* gene family; (**b**) interspecies collinearity analysis of PR1 family. Note: The *Oryza sativa*, *Triticum aestivum*, *Nicotiana benthamiana* and *Arabidopsis thaliana* are shown in different color boxes and labeled.

To explore the evolutionary process of the *TaPR1* gene family, the intraspecies collinearity (shown in Figure 4a) and interspecies collinearity (shown in Figure 4b) were analyzed. The intraspecies collinearity analysis detected 38 pairs of collinear genes among the 83 *TaPR1* genes. The results showed that all genes exhibited collinearity within homologous chromosomes, with most of them being alleles on these chromosomes and highly conserved in evolution. It can be inferred that the evolution of TaPR1 was influenced by segmental duplication.

The interspecies collinearity further revealed the evolutionary relationship between the PR1 family and other species. As shown in Figure 4b, there were eight pairs of collinear genes between *T. aestivum* L. and *O. sativa* L., but only one pair of collinear genes was found between *T. aestivum* L. and *A. thaliana* or *N. benthamiana*. According to this analysis, the PR1 members between *T. aestivum* L. and *O. sativa* L. showed high consistency and were more closely related compared to those in *A. thaliana* and *N. benthamiana*.

### 2.6. Analysis of RNA-Seq Data: Quality Assessment and Repeat Correlation

**Figure 5:**
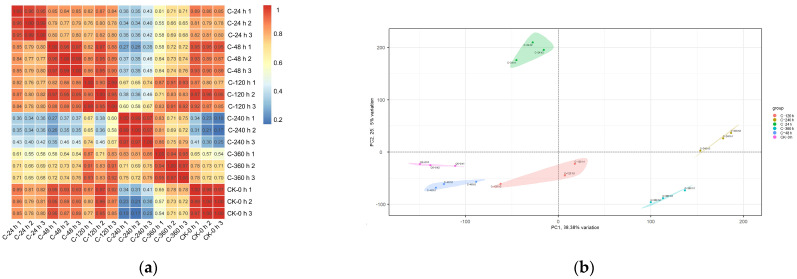
(**a**) Individual correlation analyses between 18 transcriptome samples; (**b**) PCA analysis of all samples.

Each sample produced an average of 9.78 Gb of data, with the percentage of Q30 bases exceeding 97.67%. The data accession number was PRJNA1203070, and the detailed sequencing data quality is shown in Table S4. Pearson correlation coefficients were used to construct a correlation heatmap for the samples, as shown in Figure 5a. In the control group, the heatmap color consistency among the biological replicates indicated a strong correlation across all treatment groups. PCA analysis demonstrated high repeatability among the samples, as shown in Figure 5b. The quality of 18 samples indicated that they were suitable for follow-up research.

### 2.7. GO and KEGG Enrichment Analysis of DEGs

Genes with FDR < 0.05 and |log_2_FC| > 1 were considered DEGs. The treatment groups (24 hpi, 48 hpi, 120 hpi, 240 hpi, 360 hpi) were compared with the control group (0 hpi) to elucidate the up- and downregulation patterns of the DEGs over different periods. A total of 80,750 DEGs were identified, with 40,227 significantly upregulated and 40,523 downregulated, as detailed in Table S5.

**Figure 6:**
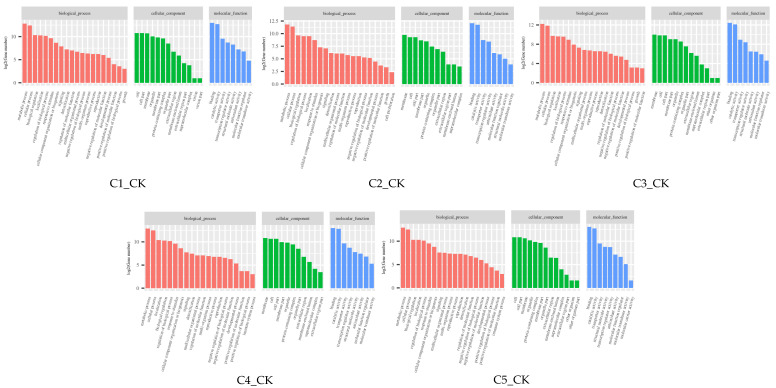
GO analysis of the DEGs in different periods. Note: C1: 24 hpi, C2: 48 hpi, C3: 120 hpi, C4: 240 hpi, C5: 360 hpi, CK: 0 hpi.

A GO analysis was conducted to assess the function of the DEGs. As shown in Figure 6, within the biological processes category, a large number of DEGs were enriched in metabolic and cellular processes across all five stages. In the cellular component category, the DEGs in the C1_CK and C5_CK stages were heavily enriched in the cell and cell parts. In the C2_CK, C3_CK, and C4_CK stages, the DEGs were mainly enriched in the membrane and cell. In the molecular function category, the DEGs were mainly enriched in binding and catalytic activity in the five stages.

**Figure 7:**
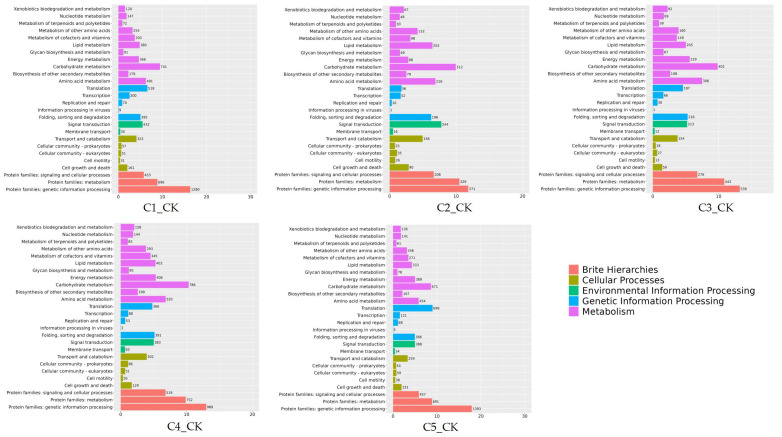
KEGG analysis of the DEGs in different periods. Note: C1: 24 hpi, C2: 48 hpi, C3: 120 hpi, C4: 240 hpi, C5: 360 hpi, CK: 0 hpi.

A KEGG pathway enrichment analysis was performed on the DEGs in the five developmental stages. The KEGG classifications were divided into five categories: brite hierarchies, cellular processes, environmental information processing, genetic information processing, and metabolism (Figure 7). In the metabolism category, most DEGs were enriched in carbohydrate metabolism in all five stages, with the C4_CK period exhibiting the most abundant (786 DEGs). In the genetic information processing category, the DEGs were mainly enriched in translation, folding, sorting, and degradation in the five stages, with translation being the most enriched in C5_CK. In the environmental information processing category, all DEGs were enriched in signal transduction. In the cellular processes category, the DEGs were mainly enriched in transport and catabolism. In the brite hierarchies category, the DEGs were distributed in all three categories, with protein families related to genetic information processing being the most predominant. GO and KEGG enrichment analysis provides valuable insights into the roles and interrelationships of genes and proteins in biological processes, highlighting their potential biological significance. This approach serves as an effective method for the rapid identification and functional determination of target genes.

### 2.8. The Results of qRT-PCR Analysis

**Figure 8:**
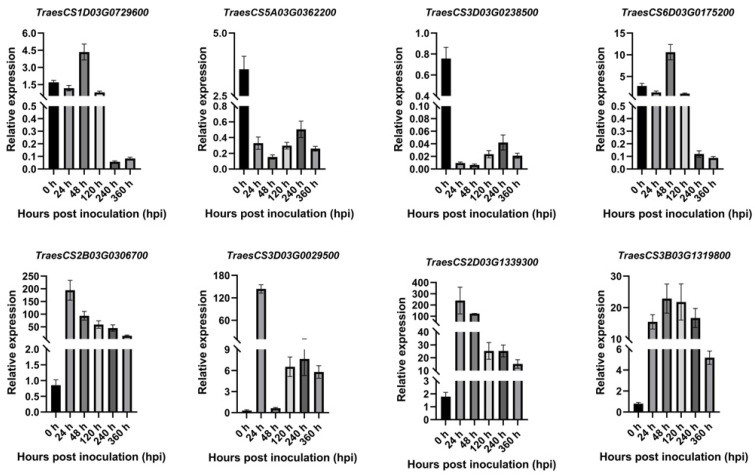
Validation of RNA-seq data using qRT-PCR. TraesCS1D03G0729600, TraesCS5A03G0362200, TraesCS3D03G0238500, and TraesCS6D03G0175200 are upregulated genes; TraesCS2B03G0306700, TraesCS3D03G0029500, TraesCS2D03G1339300, and TraesCS3B03G1319800 are downregulated genes. The image was created by GraphPad Prism 9.5.

To verify the accuracy of the RNA-seq data, four upregulated and four downregulated genes were randomly selected for qPT-PCR. The primers are shown in Table S6, and 26S was used as the internal reference. The results (Figure 8) showed that the RNA-seq data were consistent with the overall trend observed in the qRT-PCR data, confirming the accuracy and reliability of the RNA-seq results.

### 2.9. Expression Profiles of PR1 in Wheat in Response to Pst

**Figure 9:**
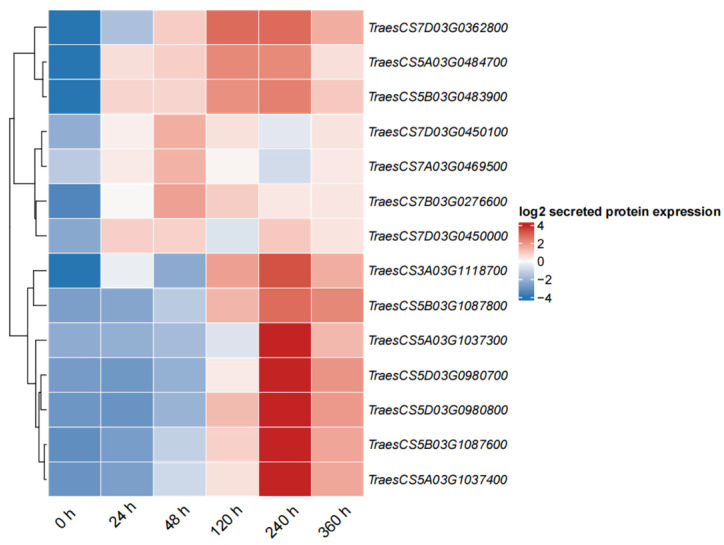
Expression heatmap of 14 *TaPR1* genes in the transcriptome. Note: The image shows the expression profiles of 14 *TaPR1* genes in XC 32 at six time points after inoculation with *Pst*.

Previously, 83 *TaPR1* genes were identified, and the distribution and expression of all *TaPR1* genes were analyzed in the XC 32 transcriptome data. Fourteen *TaPR1* genes (*TraesCS5A03G1037300*, *TraesCS5A03G1037400*, *TraesCS5A03G0484700*, *TraesCS5B03G1087600*, *TraesCS5B03G1087800*, *TraesCS5D03G0980800*, *TraesCS5D03G0980700*, *TraesCS7A03G0469500*, *TraesCS7B03G0276600*, *TraesCS5B03G0483900*, *TraesCS7D03G0450000*, *TraesCS7D03G0362800*, *TraesCS7D03G0450100*, *TraesCS3A03G1118700*) were expressed in the XC 32 transcriptome, displaying different expression patterns at various stages, as shown in Figure 9.

**Figure 10:**
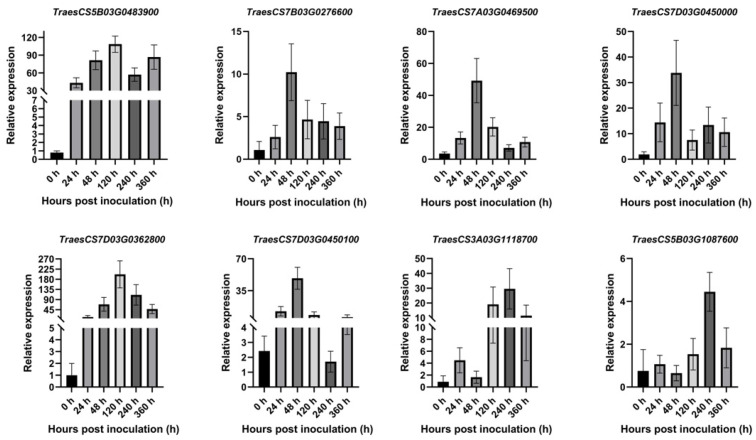
The expression profiles of 8 *TaPR1* genes in response to *Pst*. The image was created by GraphPad Prism 9.5.

To investigate the response characteristics of the PR1 family members to *Pst*, eight genes were randomly selected, and their expression levels were detected by RT-PCR. As shown in Figure 10, compared with 0 h, all TaPR1 genes at 24 hpi, 48 hpi, 120 hpi, 240 hpi, and 360 hpi were upregulated. Among them, *TraesCS5B03G0483900* and *TraesCS7D03G0362800* were not expressed at 0 hpi and were induced after infection with *Pst*. *TraesCS7B03G0276600*, *TraesCS7A03G0469500*, *TraesCS7D03G0450000*, and *TraesCS7D03G0450100* were highly expressed at 48 hpi. *TraesCS5B03G0483900* was highly expressed at 120 hpi, while *TraesCS3A03G1118700* and *TraesCS5B03G1087600* were highly expressed at 240 hpi. During the different stages of *Pst* infection, the *TaPR1* genes exhibited different expression patterns. Based on the above analysis, it can be speculated that the *TaPR1* genes were significantly expressed between 48 hpi and 240 hpi and began to decrease after 360 hpi.

## 3. Discussion

Wheat’s resistance to *Puccinia striiformis* f. sp. *tritici* (*Pst*) infection involves the coordinated regulation of multiple genes, with many genes associated with stripe rust resistance already identified. Among these, the PR1 family plays a pivotal role in regulating immune function, as demonstrated in *Arabidopsis* [16]. However, few studies have explored the regulation of PR1 family members in wheat stripe rust resistance. The accurate identification and analysis of PR1 family genes could provide a reliable foundation for the screening of candidate *TaPR1* genes involved in defense regulation. A previous study by Liu et al. [15] identified 86 *TaPR1* genes in wheat using an earlier version (the IWGSC RefSeq V1.1), which contained gaps and assembly inaccuracies in repetitive sequences and complex regions, potentially missing some important genes. The latest version of the wheat genome, IWGSC RefSeq V2.1 (released in 2020) [14], provides more comprehensive information and more complete annotations compared to the earlier V1.1 version. This updated genome is a valuable resource for the precise characterization of the *TaPR1* family. In this study, a total of 83 *TaPR1* genes were identified, which was three fewer than the number reported by Liu et al. [15], using the V1.1 wheat genome. However, a bidirectional comparison revealed discrepancies in gene annotation between the two genome versions. Multiple sequence alignments suggested that these discrepancies stemmed from missplicing or misannotation in the 2018 genome version. This underscores the importance of further studies utilizing the IWGSC RefSeq V2.1 data. Our analysis also revealed that the previous dataset included redundant gene entries, highlighting the improved accuracy and completeness of the V2.1 annotations. These findings provide a more reliable and comprehensive understanding of the *TaPR1* gene family and reinforce the value of the IWGSC RefSeq V2.1 dataset for future research.

The PR1 family exhibits conservation across different species. Comparative studies in tomato [17], *Arabidopsis*, and rice [18] have similarly identified conserved CAP domains in PR1 family members, supporting the concept of evolutionary conservation across species. In the present study, the results indicated that the *TaPR1* family was highly conserved, as shown in Figure 1, with a complete CAP-PR1 domain. The CAP domain is a hallmark feature of PR1 proteins and plays a critical role in their biological functions, such as plant defense and stress responses. All *TaPR1* genes identified in this study shared a conserved CAP domain and motifs (motifs 1, 2, 3, 4, 5, 6, and 7), as shown in Figure 1, suggesting functional conservation across the gene family. Interestingly, only three *TaPR1* genes contained the CAP domain alongside the STKt_IRAK, PRK10263 superfamily, and PspC_subgroup_2 superfamily domains, indicating that these genes might have specialized roles in unique biological processes. These additional domains might contribute to specific regulatory roles or interactions, distinguishing these genes from other PR1 family members. This suggests that the functional roles of PR1 proteins in plant immunity have been maintained throughout evolution, likely reflecting their critical importance in defense mechanisms. Phylogenetic (Figure 2) and collinearity analyses (Figure 4) further revealed that the *TaPR1* genes in wheat are more closely related to those in rice than to their counterparts in *Arabidopsis*. This closer evolutionary relationship between the wheat and rice PR1 genes could be attributed to their shared monocot lineage, whereas *Arabidopsis*, as a dicot, diverged earlier during plant evolution. Such phylogenetic insights provide valuable information about the conservation and diversification of PR1 genes, highlighting the relevance of monocot-specific adaptations in plant defense systems. Moreover, identifying conserved domains and motifs across *TaPR1* genes not only enhanced our understanding of their structural and functional properties but also lays a foundation for further functional studies. These findings suggest that specific domain combinations, such as those found in the three unique *TaPR1* genes, might be involved in specialized defense responses, potentially tailored to combat specific pathogens or environmental stresses. This study underscores the importance of the domain architecture and evolutionary relationships in understanding the functional diversity of PR1 family members across plant species.

Plants respond to pathogen infection by activating defense mechanisms, where local and systemic acquired resistance is triggered when host-encoded resistance genes recognize pathogen-encoded avirulence genes. Salicylic acid (SA) is an important signal molecule involved in activating defense-related proteins and regulating disease progression [17]. The expression of the PR1 members has been widely recognized as a reliable marker for SA-mediated defense responses and the establishment of acquired resistance [18,19]. The transcription level of PR1 has been extensively used as a marker for disease resistance [20], and the significant accumulation of PR1 transcripts has also been observed in wheat lines resistant to *Fusarium graminearum* and *Mycosphaerella graminicola* [21]. Li et al. reported that the wheat PR1 protein played a role in the Lr35-mediated adult plant resistance to leaf rust caused by *Pst* [22]. However, no reports have directly shown that the presence of TaPR1 affects pathogens or pathogen dynamics [23]. Therefore, 83 *TaPR1* genes were identified and analyzed regarding their expression patterns in the wheat–Pst interaction in XC 32 to predict the actual function of the *TaPR1* gene. PR1’s protein-like gene expression was upregulated in both resistant and susceptible genotypes of tomato under *Alternaria solani* infection [24]. This was similar to the results of this study, where the RNA-seq analysis showed that approximately 14 *TaPR1* genes were upregulated in XC 32, as shown in Figure 9, with varying expression patterns during different periods of *Pst* infection. Among them, *TraesCS5A03G1037300*, *TraesCS5A03G1037400*, *TraesCS5B03G1087600*, *TraesCS5B03G1087800*, *TraesCS5D03G0980800*, *TraesCS5D03G0980700*, and *TraesCS7A03G0469500* were induced by *Pst* and began to be expressed at 48 hpi. Systemic acquired resistance (SAR) mediated by salicylic acid (SA) is a broad-spectrum mechanism of plant resistance, with NPR1 and TGA as the main regulatory factors [25]. From the transcriptome data in this study, the expression of the NPR1 and TGA genes increased 24 h after *Pst* inoculation. Previous studies have shown that [25,26,27] the high expression of *PR1* leads to lignin accumulation, which activates the SA and JA pathways, thereby increasing plants’ resistance to pathogens. This idea can be used to verify the mechanism of *TaPR1*’s interaction with *Pst.* Additionally, the transcriptome data and qPCR analysis showed that most *TaPR1* family members, as shown in Figure 10, were highly expressed at 24 hpi; they were initially expressed at low levels, with their expression increasing over time. This expression pattern suggests a possible positive regulatory role. In our previous study, the transcriptome expression of the susceptible variety MX 169 in conjunction with the XC 32 transcriptome data was analyzed, and we found that the *TaPR1-66* gene was significantly upregulated 24 h after inoculation with *Pst*. However, this study still has its limitations, as it only identified the *TaPR1* genes that were significantly expressed during the wheat–*Pst* interaction and determined a candidate gene without further validation. This will be addressed in future work by verifying the gene function and screening interacting proteins using LC-MS/MS to clarify the interaction mechanism with *Pst*.

## 4. Materials and Methods

### 4.1. Identification and Comparison of PR1

The protein sequences from wheat (*Triticum aestivum* L.), rice (*Oryza sativa*, cv. Nipponbare), *Arabidopsis* (*Arabidopsis thaliana*), and tobacco (*Nicotiana benthamiana*) were downloaded from Ensembl Plants (https://plants.ensembl.org/info/data/ftp/index.html (accessed on 15 April 2024)), and the wheat genome sequences, including DNA FASTA and GFF3 files, were downloaded from URGI (https://urgi.versailles.inra.fr/download/iwgsc/IWGSC_RefSeq_Assemblies/v2.1/, https://urgi.versailles.inra.fr/download/iwgsc/IWGSC_RefSeq_Annotations/v2.1/ (accessed on 20 April 2024)). The PR1 protein sequences from various known cultivars were collected and used as query sequences for the initial BLAST search (e-value < 10^−10^) within the IWSGC genomes using TBtools (V2.119) [28]. Redundant sequences were manually discarded, and the results from the initial BLAST search were employed as queries for a second BLAST search (e-value < 10^−10^) [29]. The results were then compared, and non-PR1 members were removed. The protein sequences identified from the two BLAST searches were analyzed for the conserved PR1 domain using the online NCBI-CDD (Conserved Domain Database, https://www.ncbi.nlm.nih.gov/cdd (accessed on 1 May 2024)) [30].

Liu et al., 2023 identified and downloaded the PR1 gene sequences from the IWGSC RefSeq V1.1 [15]. In our study, these sequences were used as queries for a BLAST search in the IWGSC RefSeq V2.1 to identify the differences in the wheat PR1 family in the two versions. The PR1 genes identified in the different versions of the wheat genome were analyzed using forward and reverse BLAST. SnapGene was utilized to perform multiple alignments of the uncertain genes. Genes differentially identified in previous studies and the current research were employed for BLAST searches within the Chinese Spring genome [29].

### 4.2. Gene Structure and Phylogenetic Analysis

The coding sequence of each *TaPR1* gene was aligned with its genomic sequence using TBtools (V 2.119) to construct a gene structure map. To identify the evolutionary relationships between the *TaPR1* proteins from wheat, *Arabidopsis*, tobacco, and rice, all of the amino acid sequences were aligned using Clustal W in MEGA X (V 10.1.7). The phylogenetic tree was constructed using the neighbor-joining method, based on the WAG + G matrix-based model with 1000 bootstrap replications.

### 4.3. Cis-Acting Element (CRE), Conserved Motif, and Conserved Domain Analysis

Promoter regions were defined as sequences 1500 bp upstream of the start codons. The 2000 bp sequences upstream of the start codons were extracted using TBtools from the genome sequences and submitted for CRE prediction using the online software PlantCARE (http://bioinformatics.psb.ugent.be/webtools/plantcare/html (accessed on 17 May 2024)) [31].

The motifs of all PR1 protein sequences were identified using the online software MEME 5.5.7 (Multiple EM for Motif Elicitation, https://meme-suite.org/meme/tools/meme (accessed on 25 May 2024)). The parameter settings were as follows: number of repetitions—any, maximum number of motifs—20, and optimum motif width ≥ 6 and ≤200 [32]. Conserved domains within the PR1 family were identified using the Pfam database, with confirmation from the NCBI Conserved Domain Database (https://www.ncbi.nlm.nih.gov/Structure/bwrpsb/bwrpsb.cgi (accessed on 30 May 2024)).

### 4.4. Chromosomal Localization and Collinearity Analysis

All identified *TaPR1* genes were analyzed by aligning their sequences with the corresponding genome reference GFF3 file (IWGSC V 2.1) to extract the start and end location information of the genes. The software TBtools (https://github.com/CJ-Chen/TBtools-II/releases (accessed on 5 June 2024)) [28] was used for visualization to determine their chromosomal locations. Interspecies and intraspecies collinearity were analyzed using the Multicollinearity Scanning Toolkit X [33] and visualized with TBtools (V 2.119).

### 4.5. Plant and Fungal Materials

The wheat varieties Xinchun 32 (XC 32), Minxian 169 (MX 169), and *Pst* were obtained from the Plant Epidemic Laboratory at the College of Agriculture of Xinjiang Agricultural University. XC 32 was developed in Xinjiang and exhibits high resistance to the currently epidemic *Pst* races (CYR32, CYR34). The *Pst* was expanded and preserved by MX 169. Mixed *Pst* was artificial inoculated at the two-leaf stage of wheat. The inoculated wheat was placed in an artificial incubator at the Plant Epidemic Laboratory, incubated at 80% humidity and 11 °C in the dark for 24–36 h, and then transferred to 148–185 μmol photon m^−2^s^−1^ light intensity with a 16 h light/8 h dark cycle. Wheat leaves were collected at 0 h, 12 h, 24 h, 48 h, 120 h, 240 h, and 360 h after inoculation with *Pst*, with 3 biological replicates at each time point. All leaf samples were frozen in liquid nitrogen and stored at −80 °C for subsequent RNA-seq.

### 4.6. RNA-Sequencing, Kyoto Encyclopedia of Genes and Genomes Analysis, and Gene Ontology Analysis

The RNA extraction, library construction, and transcriptome sequencing and analysis were conducted by the Wuhan Igenbook Biotechnology Company (Wuhan, China). Based on the raw data, adapter, Poly-N, and low-quality reads were removed to obtain clean reads, ensuring the accuracy of the subsequent analysis. All clean reads were aligned to the reference genome (IWGSC RefSeq V2.1) using Hisat2 (version: 2.0.1-beta). The read counts for each gene were obtained with FeatureCounts (version: v1.6.0), and the fragments per kilobase of exon per million reads mapped (FPKM) for each gene was calculated. Differentially expressed genes (DEGs) among different samples were analyzed by the R package; the DEGs were identified according to FDR < 0.05 and |log2 FC| > 1. KEGG and GO analyses of the DEGs were performed using the R package (V 3.10.1).

### 4.7. qRT-PCR Analysis

Total RNA was extracted using the RNAprep Pure Plant Total RNA Extraction Kit (TIANGEN, DP432, Beijing, China). First-strand cDNA synthesis was carried out using the ABScrip III RT Master Mix for qPCR with gDNA Remover (ABclonal, Woburn, MA, USA). The primers used for the qRT-PCR analyses were designed based on gene sequences identified by Beacon Designer 8.14.

The qRT-PCR was conducted using 2× Universal SYBR Green Fast qPCR Mix (ABclonal) on a CFX96 Real-Time System (Bio Rad, Hercules, CA, USA). All genes were detected using three biological replicates and three technological replicates. The 26S gene was used as an internal reference to normalize the relative expression of the candidate genes, and the mRNA expression levels were calculated using the 2^−ΔΔCt^ method [34].

## 5. Conclusions

In this study, the *TaPR1* gene family was comprehensively analyzed using the wheat genome IWGSC RefSeq V2.1, and we identified the *TaPR1* genes involved in the wheat–*Pst* interaction from the XC 32 transcriptome. A total of 83 *TaPR1* genes were identified. Fourteen *TaPR1* genes involved in the wheat–*Pst* interaction were identified in the XC 32 transcriptome. The *TaPR1* gene family was characterized by analyzing the conserved domains and motifs, chromosome distribution, phylogeny, gene structure, and collinearity. The results showed that all *TaPR1* genes contained the same motifs (motifs 1, 2, 3, 4, 5, 6, and 7) and CAP domain. Only three genes contained the CAP domain along with the STKt_IRAK, PRK10263 superfamily, and PspC_subgroup_2 superfamily domains. These results indicate that the genes were highly similar in function and showed highly conserved sequences. The phylogenetic and collinearity analyses showed that wheat and rice are more closely related to each other than to *Arabidopsis* and tobacco. Additionally, wheat samples were collected at different time points post-*Pst* inoculation for qRT-PCR analysis. The results showed that, compared to 0 hpi, the *TaPR1* gene was significantly upregulated after *Pst* inoculation, with *Pst* infection inducing the high expression of the *TaPR1* gene in wheat. These findings provide valuable insights into the evolutionary characteristics of the *TaPR1* gene in wheat.

## Data Availability

The data are contained within the article.

## Supplementary Materials

The following supporting information can be downloaded at: https://www.mdpi.com/article/10.3390/ijms26020640/s1.
