# Supplementary material for: Re-Examination Characterization and Screening of Stripe Rust Resistance Gene of Wheat TaPR1 Gene Family Based on the Transcriptome in Xinchun 32
Source: Int J Mol Sci. 2025 Jan 14;26(2):640. doi: 10.3390/ijms26020640 (PMC11766189; doi:10.3390/ijms26020640)
Supplement: Supplementary file 1 [file ijms-26-00640-s001.zip › Table S1.pdf]

Table S1. Gene renaming file.

No.	Gene ID	Rename ID
1	*TraesCS1A03G1081700.1*	*TaPR1-01*
2	*TraesCS1A03G1081400.1*	*TaPR1-02*
3	*TraesCS1B03G1278500.1*	*TaPR1-03*
4	*TraesCS1B03G1279100.1*	*TaPR1-04*
5	*TraesCS1D03G1042600.1*	*TaPR1-05*
6	*TraesCS2A03G1046500.1*	*TaPR1-06*
7	*TraesCS2A03G1043900.1*	*TaPR1-07*
8	*TraesCS2A03G1043600.1*	*TaPR1-08*
9	*TraesCS2B03G1163900.1*	*TaPR1-09*
10	*TraesCS2B03G1164100.1*	*TaPR1-10*
11	*TraesCS2B03G1163800.1*	*TaPR1-11*
12	*TraesCS2B03G1028200.1*	*TaPR1-12*
13	*TraesCS2B03G1028100.1*	*TaPR1-13*
14	*TraesCS2D03G0981400.1*	*TaPR1-14*
15	*TraesCS2D03G0981700.1*	*TaPR1-15*
16	*TraesCS2D03G0982100.1*	*TaPR1-16*
17	*TraesCS2D03G0873100.1*	*TaPR1-17*
18	*TraesCS2D03G0981800.1*	*TaPR1-18*
19	*TraesCS2D03G0981600.1*	*TaPR1-19*
20	*TraesCS2D03G0981900.1*	*TaPR1-20*
21	*TraesCS3A03G1240400.1*	*TaPR1-21*
22	*TraesCS3D03G1172200.1*	*TaPR1-22*
23	*TraesCS5A03G1037300.1*	*TaPR1-23*
24	*TraesCS5A03G0029400.1*	*TaPR1-24*
25	*TraesCS5A03G1037400.1*	*TaPR1-25*
26	*TraesCS5A03G0484700.1*	*TaPR1-26*
27	*TraesCS5A03G1038000.1*	*TaPR1-27*
28	*TraesCS5A03G0150600.1*	*TaPR1-28*
29	*TraesCS5A03G1037800.1*	*TaPR1-29*
30	*TraesCS5B03G1089500.1*	*TaPR1-30*
31	*TraesCS5B03G1090200.1*	*TaPR1-31*
32	*TraesCS5B03G1089100.1*	*TaPR1-32*
33	*TraesCS5B03G1087600.1*	*TaPR1-33*
34	*TraesCS5B03G1089700.1*	*TaPR1-34*
35	*TraesCS5B03G1088800.1*	*TaPR1-35*
36	*TraesCS5B03G1089600.1*	*TaPR1-36*
37	*TraesCS5B03G1087800.1*	*TaPR1-37*
38	*TraesCS5B03G1090100.1*	*TaPR1-38*
39	*TraesCS5B03G1088200.1*	*TaPR1-39*
40	*TraesCS5B03G0163000.1*	*TaPR1-40*
41	*TraesCS5B03G0024200.1*	*TaPR1-41*

Table S1. *Cont.*

No.	Gene ID	Rename ID
42	*TraesCS5B03G1089800.1*	*TaPR1-42*
43	*TraesCS5B03G1088900.1*	*TaPR1-43*
44	*TraesCS7B03G0276600.1*	*TaPR1-44*
45	*TraesCS5B03G1090000.1*	*TaPR1-45*
46	*TraesCS5D03G0980800.1*	*TaPR1-46*
47	*TraesCS5D03G0981900.1*	*TaPR1-47*
48	*TraesCS5D03G0980700.1*	*TaPR1-48*
49	*TraesCS5D03G0981000.1*	*TaPR1-49*
50	*TraesCS6A03G0890500.1*	*TaPR1-50*
51	*TraesCS6A03G0892900.1*	*TaPR1-51*
52	*TraesCS6A03G0890300.1*	*TaPR1-52*
53	*TraesCS6A03G0890400.1*	*TaPR1-53*
54	*TraesCS6B03G1074200.1*	*TaPR1-54*
55	*TraesCS6B03G1069000.1*	*TaPR1-55*
56	*TraesCS6B03G1069600.1*	*TaPR1-56*
57	*TraesCS6B03G1069200.1*	*TaPR1-57*
58	*TraesCS6D03G0765100.1*	*TaPR1-58*
59	*TraesCS6D03G0765000.1*	*TaPR1-59*
60	*TraesCS6D03G0767200.1*	*TaPR1-60*
61	*TraesCS6D03G0765300.1*	*TaPR1-61*
62	*TraesCS7A03G1378300.1*	*TaPR1-62*
63	*TraesCS7A03G0355500.1*	*TaPR1-63*
64	*TraesCS7A03G0469300.1*	*TaPR1-64*
65	*TraesCS7A03G0469500.1*	*TaPR1-65*
66	*TraesCS5B03G0483900.1*	*TaPR1-66*
67	*TraesCS7B03G0275300.1*	*TaPR1-67*
68	*TraesCS7B03G0276300.1*	*TaPR1-68*
69	*TraesCS7B03G0275900.1*	*TaPR1-69*
70	*TraesCS7B03G0151100.1*	*TaPR1-70*
71	*TraesCS7B03G0275500.1*	*TaPR1-71*
72	*TraesCS7D03G0450000.1*	*TaPR1-72*
73	*TraesCS7D03G0226600.1*	*TaPR1-73*
74	*TraesCS7D03G0362800.1*	*TaPR1-74*
75	*TraesCS7D03G0343800.1*	*TaPR1-75*
76	*TraesCS7D03G0450100.1*	*TaPR1-76*
77	*TraesCSU03G0404500.1*	*TaPR1-77*
78	*TraesCSU03G0385100.1*	*TaPR1-78*
79	*TraesCSU03G0309900.1*	*TaPR1-79*
80	*TraesCS3A03G1118700.1*	*TaPR1-80*
81	*TraesCS4A03G0659100.1*	*TaPR1-81*
82	*TraesCS4B03G0138400.1*	*TaPR1-82*
83	*TraesCS4D03G0116900.1*	*TaPR1-83*
