# Supplementary material for: Re-Examination Characterization and Screening of Stripe Rust Resistance Gene of Wheat TaPR1 Gene Family Based on the Transcriptome in Xinchun 32
Source: Int J Mol Sci. 2025 Jan 14;26(2):640. doi: 10.3390/ijms26020640 (PMC11766189; doi:10.3390/ijms26020640)
Supplement: Supplementary file 1 [file ijms-26-00640-s001.zip › Table S2.pdf]

Table S2. List of physicochemical properties of *TaPR1* gene in wheat.

No.	Sequence ID	Number of Amino Acid(aa)	Molecular Weight (kDa)	Theoretical pI	Instability Index	Aliphatic Index	Grand Average of Hydropathicity
1	*TaPR1-01*	244	26.91746	8.82	49.69	63.24	-0.443
2	*TaPR1-02*	294	30.4655	5.73	71.19	50.37	-0.449
3	*TaPR1-03*	218	23.69119	6.05	55.37	56.1	-0.462
4	*TaPR1-04*	245	26.91943	8.7	52.24	66.57	-0.33
5	*TaPR1-05*	245	26.85242	8.96	47.98	63.39	-0.394
6	*TaPR1-06*	183	19.9245	8.66	44.91	70.93	-0.201
7	*TaPR1-07*	181	19.74838	8.79	54.99	70.72	-0.339
8	*TaPR1-08*	157	16.87788	6.05	65.24	74.65	-0.183
9	*TaPR1-09*	181	19.54522	8.8	54.17	75.03	-0.214
10	*TaPR1-10*	183	20.02757	9.03	43.35	68.25	-0.371
11	*TaPR1-11*	179	19.42982	5.23	56.6	76.93	-0.16
12	*TaPR1-12*	176	19.09977	8.73	45.98	79.38	-0.091
13	*TaPR1-13*	191	20.6742	8.11	55.3	69.53	-0.32
14	*TaPR1-14*	159	17.02796	6.04	68.2	71.26	-0.216
15	*TaPR1-15*	183	19.89431	5.86	61.38	74.75	-0.197
16	*TaPR1-16*	185	20.22179	9.17	43.22	65.95	-0.343
17	*TaPR1-17*	194	21.01093	8.73	54.02	77.06	-0.143
18	*TaPR1-18*	181	19.75425	8.41	49.51	68.01	-0.348
19	*TaPR1-19*	183	19.74708	5.26	61.68	73.66	-0.177
20	*TaPR1-20*	181	19.63834	9.08	52.49	73.37	-0.248
21	*TaPR1-21*	165	17.65209	4.85	32.25	87.64	0.104
22	*TaPR1-22*	165	17.59598	4.85	34.39	85.21	0.093
23	*TaPR1-23*	166	17.83149	4.4	42.54	65.84	-0.34
24	*TaPR1-24*	312	33.63358	5.14	57.74	59.71	-0.503
25	*TaPR1-25*	167	17.80164	4.82	39.63	70.12	-0.265
26	*TaPR1-26*	164	17.56578	8.52	26.9	72.07	-0.226
27	*TaPR1-27*	166	17.79903	5.85	36.59	78.86	-0.032
28	*TaPR1-28*	164	17.74899	7.61	33.86	73.35	-0.158
29	*TaPR1-29*	166	17.5583	4.47	37.78	68.19	-0.16
30	*TaPR1-30*	165	17.75845	4.28	32.49	65.64	-0.289
31	*TaPR1-31*	165	17.81053	4.42	34.98	65.64	-0.29
32	*TaPR1-32*	168	17.8815	4.28	35.66	64.46	-0.312
33	*TaPR1-33*	166	17.70237	4.47	41.96	64.1	-0.33
34	*TaPR1-34*	165	17.77445	4.28	35.15	65.03	-0.289
35	*TaPR1-35*	168	17.92157	4.28	35.16	64.46	-0.32
36	*TaPR1-36*	165	17.80053	4.28	32.49	67.39	-0.261
37	*TaPR1-37*	185	19.86814	5.18	48.71	73.84	-0.186
38	*TaPR1-38*	165	17.77445	4.28	35.15	65.03	-0.289
39	*TaPR1-39*	165	17.55013	4.28	33.47	64.48	-0.296
40	*TaPR1-40*	164	17.62679	8.52	27.52	71.59	-0.221

Table S2. *Cont.*

No.	Sequence ID	Number of Amino Acid(aa)	Molecular Weight (kDa)	Theoretical pI	Instability Index	Aliphatic Index	Grand Average of Hydropathicity
41	*TaPR1-41*	304	32.9547	5.25	59.8	60.03	-0.542
42	*TaPR1-42*	165	17.80053	4.28	32.49	67.39	-0.261
43	*TaPR1-43*	158	16.89151	4.38	37.5	66.71	-0.304
44	*TaPR1-44*	174	18.79713	7.63	25.11	71.26	-0.142
45	*TaPR1-45*	188	20.14622	4.23	34.87	74.68	-0.067
46	*TaPR1-46*	167	17.81864	4.58	38.89	69.58	-0.259
47	*TaPR1-47*	168	18.0703	6.26	35.32	77.26	-0.079
48	*TaPR1-48*	166	17.73244	4.47	40.35	67.59	-0.283
49	*TaPR1-49*	168	17.86747	4.26	34.69	64.46	-0.282
50	*TaPR1-50*	213	22.79468	7.05	48.58	72.21	-0.083
51	*TaPR1-51*	179	19.04685	8.92	45.18	71.68	-0.02
52	*TaPR1-52*	184	19.328	10.41	36.7	70.82	-0.168
53	*TaPR1-53*	174	18.91542	8.83	37.4	66.9	-0.111
54	*TaPR1-54*	179	19.08086	9.05	44.35	69.5	-0.035
55	*TaPR1-55*	188	19.81576	10.37	41.4	73.4	-0.105
56	*TaPR1-56*	213	22.84093	8.03	48.03	75.35	0.006
57	*TaPR1-57*	177	19.16268	8.83	37.21	65.2	-0.106
58	*TaPR1-58*	177	19.17267	8.83	41.29	65.25	-0.135
59	*TaPR1-59*	188	19.82871	10.29	40.56	73.94	-0.078
60	*TaPR1-60*	179	19.14791	8.73	47.59	68.94	-0.035
61	*TaPR1-61*	213	22.84579	7.11	49.45	70.38	-0.11
62	*TaPR1-62*	167	18.37472	9.86	41.09	67.19	-0.375
63	*TaPR1-63*	174	18.77615	6.93	42.79	70.29	-0.196
64	*TaPR1-64*	172	18.61787	9.02	23.78	65.29	-0.267
65	*TaPR1-65*	174	18.89819	7.63	24.49	69.02	-0.207
66	*TaPR1-66*	164	17.63489	8.74	27.57	72.07	-0.236
67	*TaPR1-67*	174	18.83518	9.17	25.75	67.93	-0.207
68	*TaPR1-68*	174	18.85015	9.02	25.92	65.11	-0.237
69	*TaPR1-69*	174	18.83612	9.02	27.03	65.11	-0.238
70	*TaPR1-70*	172	18.6181	8.46	43.63	70	-0.295
71	*TaPR1-71*	163	17.93831	11.62	44.94	70.67	-0.554
72	*TaPR1-72*	172	18.65897	9.17	28.92	66.4	-0.273
73	*TaPR1-73*	169	18.16437	7.53	27.47	69.35	0.03
74	*TaPR1-74*	164	17.53674	8.75	28.34	72.13	-0.218
75	*TaPR1-75*	172	18.60899	8.14	45.49	69.42	-0.34
76	*TaPR1-76*	173	18.80004	6.88	25.96	68.84	-0.201
77	*TaPR1-77*	168	17.8815	4.28	35.66	64.46	-0.297
78	*TaPR1-78*	168	17.89459	4.4	35.92	65.06	-0.286
79	*TaPR1-79*	168	17.8815	4.28	35.66	64.46	-0.297
80	*TaPR1-80*	747	80.78124	6.17	40.29	80.86	-0.236
81	*TaPR1-81*	185	19.22569	9.36	42.21	67.95	-0.132

82	*TaPR1-82*	185	19.07546	9.1	37.87	68.49	-0.056
83	*TaPR1-83*	183	19.02952	9.24	40.31	71.86	-0.063
