# Supplementary material for: Re-Examination Characterization and Screening of Stripe Rust Resistance Gene of Wheat TaPR1 Gene Family Based on the Transcriptome in Xinchun 32
Source: Int J Mol Sci. 2025 Jan 14;26(2):640. doi: 10.3390/ijms26020640 (PMC11766189; doi:10.3390/ijms26020640)
Supplement: Supplementary file 1 [file ijms-26-00640-s001.zip › Table S3.pdf]

Table S3. Domain prediction information of PR1 genes in wheat.

#Batch CD-search tool	NIH/NLM/NCBI						
#cdsid	QM3-qcdsearch-30F05823E0EE569D-2942E5B1AE31C4AF						
#datatype	hitsConcise Results						
#Start time: 2024-10-05T09:41:36				Run time: 0:00:00:12			
Query	Hit type	PSSM-ID	From	To	E-Value	Bitscore	Short name
Q#1 - >TaPR1-01	specific	349400	110	242	1.04028e-63	195.16	CAP_PR-1
Q#2 - >TaPR1-02	superfamily	412178	146	278	2.23438e-23	92.6968	CAP superfamily
Q#3 - >TaPR1-03	superfamily	412178	74	202	3.65355e-26	98.0896	CAP superfamily
Q#4 - >TaPR1-04	specific	349400	111	243	1.1373e-63	194.775	CAP_PR-1
Q#5 - >TaPR1-05	specific	349400	111	243	8.56416e-64	195.16	CAP_PR-1
Q#6 - >TaPR1-06	superfamily	412178	47	183	2.79277e-50	158.566	CAP superfamily
Q#7 - >TaPR1-07	superfamily	412178	47	181	2.03063e-40	133.528	CAP superfamily
Q#8 - >TaPR1-08	superfamily	412178	42	157	1.1048e-33	115.424	CAP superfamily
Q#9 - >TaPR1-09	superfamily	412178	47	181	1.9441e-40	133.528	CAP superfamily
Q#10 - >TaPR1-10	superfamily	412178	47	183	3.41942e-53	165.885	CAP superfamily
Q#11 - >TaPR1-11	superfamily	412178	46	179	4.03921e-39	130.061	CAP superfamily
Q#12 - >TaPR1-12	superfamily	412178	39	176	1.19685e-55	171.663	CAP superfamily
Q#13 - >TaPR1-13	superfamily	412178	54	191	2.39151e-54	168.966	CAP superfamily
Q#14 - >TaPR1-14	superfamily	412178	44	159	2.61843e-33	114.268	CAP superfamily
Q#15 - >TaPR1-15	superfamily	412178	50	183	1.84693e-40	133.528	CAP superfamily
Q#16 - >TaPR1-16	superfamily	412178	49	185	3.57843e-55	170.892	CAP superfamily
Q#17 - >TaPR1-17	superfamily	412178	58	194	9.29594e-57	175.515	CAP superfamily
Q#18 - >TaPR1-18	superfamily	412178	47	181	1.42508e-39	131.217	CAP superfamily
Q#19 - >TaPR1-19	superfamily	412178	50	183	4.81322e-40	132.372	CAP superfamily
Q#20 - >TaPR1-20	superfamily	412178	47	181	1.92305e-40	133.528	CAP superfamily
Q#21 - >TaPR1-21	specific	349400	27	165	5.11726e-63	190.152	CAP_PR-1
Q#22 - >TaPR1-22	specific	349400	28	165	9.14206e-63	189.382	CAP_PR-1
Q#23 - >TaPR1-23	specific	349400	27	166	3.89782e-85	246.006	CAP_PR-1
Q#24 - >TaPR1-24	specific	349400	182	312	2.37308e-74	224.435	CAP_PR-1
Q#24 - >TaPR1-24	superfamily	468202	48	149	8.55826e-05	43.9892	PspC_subgroup_2 superfamily
Q#25 - >TaPR1-25	specific	349400	28	167	6.79137e-84	242.925	CAP_PR-1
Q#26 - >TaPR1-26	specific	349400	28	164	1.51466e-79	231.754	CAP_PR-1
Q#27 - >TaPR1-27	specific	349400	27	166	4.00315e-67	200.553	CAP_PR-1
Q#28 - >TaPR1-28	specific	349400	28	164	8.92224e-75	219.813	CAP_PR-1
Q#29 - >TaPR1-29	specific	349400	28	166	1.0007e-79	232.524	CAP_PR-1

Table S3. *Cont.*

Query	Hit type	PSSM-ID	From	To	E-Value	Bitscore	Short name
Q#30 - >TaPR1-30	specific	349400	27	165	6.27394e-84	242.925	CAP_PR-1
Q#31 - >TaPR1-31	specific	349400	27	165	6.7745e-84	242.925	CAP_PR-1
Q#32 - >TaPR1-32	specific	349400	28	168	7.77638e-77	225.205	CAP_PR-1
Q#33 - >TaPR1-33	specific	349400	27	166	2.15513e-86	249.088	CAP_PR-1
Q#34 - >TaPR1-34	specific	349400	27	165	9.00944e-84	242.539	CAP_PR-1
Q#35 - >TaPR1-35	specific	349400	28	168	1.1548e-77	227.131	CAP_PR-1
Q#36 - >TaPR1-36	specific	349400	27	165	3.62688e-82	238.687	CAP_PR-1
Q#37 - >TaPR1-37	specific	349400	28	166	5.98355e-84	243.695	CAP_PR-1
Q#38 - >TaPR1-38	specific	349400	27	165	9.00944e-84	242.539	CAP_PR-1
Q#39 - >TaPR1-39	specific	349400	25	165	4.92491e-78	227.902	CAP_PR-1
Q#40 - >TaPR1-40	specific	349400	28	164	1.59121e-72	214.035	CAP_PR-1
Q#41 - >TaPR1-41	specific	349400	174	304	3.95414e-72	218.657	CAP_PR-1
Q#41 - >TaPR1-41	superfamily	236669	32	169	0.00046476	41.9943	PRK10263 superfamily
Q#42 - >TaPR1-42	specific	349400	27	165	3.62688e-82	238.687	CAP_PR-1
Q#43 - >TaPR1-43	specific	349400	28	158	2.39293e-69	205.946	CAP_PR-1
Q#44 - >TaPR1-66	specific	349400	28	164	4.94308e-87	250.629	CAP_PR-1
Q#45 - >TaPR1-45	specific	349400	50	188	3.67964e-84	244.465	CAP_PR-1
Q#46 - >TaPR1-46	specific	349400	28	167	1.03019e-83	242.539	CAP_PR-1
Q#47 - >TaPR1-47	specific	349400	27	168	1.07478e-63	192.078	CAP_PR-1
Q#48 - >TaPR1-48	specific	349400	27	166	1.31621e-85	247.162	CAP_PR-1
Q#49 - >TaPR1-49	specific	349400	28	168	2.15854e-78	229.057	CAP_PR-1
Q#50 - >TaPR1-50	specific	349400	74	213	3.20216e-62	190.152	CAP_PR-1
Q#51 - >TaPR1-51	superfamily	412178	42	179	1.46791e-36	123.513	CAP superfamily
Q#52 - >TaPR1-52	superfamily	412178	45	184	1.6968e-48	153.944	CAP superfamily
Q#53 - >TaPR1-53	specific	349400	41	174	2.8832e-78	229.057	CAP_PR-1
Q#54 - >TaPR1-54	superfamily	412178	42	179	3.35339e-36	122.357	CAP superfamily
Q#55 - >TaPR1-55	superfamily	412178	49	188	2.25297e-48	153.944	CAP superfamily
Q#56 - >TaPR1-56	specific	349400	76	213	5.98233e-61	186.686	CAP_PR-1
Q#57 - >TaPR1-57	specific	349400	44	177	1.79302e-78	229.828	CAP_PR-1
Q#58 - >TaPR1-58	specific	349400	44	177	2.06756e-78	229.443	CAP_PR-1
Q#59 - >TaPR1-59	superfamily	412178	49	188	3.29637e-47	150.862	CAP superfamily

Q#60 - >TaPR1-60	superfamily	412178	42	179	3.70193e-35	120.046	CAP superfamily
Q#61 - >TaPR1-61	specific	349400	74	213	1.23778e-62	190.923	CAP_PR-1
Q#62 - >TaPR1-62	specific	349400	29	164	2.31905e-83	241.769	CAP_PR-1
Q#63 - >TaPR1-63	superfamily	412178	26	149	1.99037e-26	97.3192	CAP superfamily
Q#64 - >TaPR1-64	specific	349400	29	164	2.36838e-89	256.792	CAP_PR-1
Q#65 - >TaPR1-65	specific	349400	29	164	7.84357e-90	258.333	CAP_PR-1
Q#66 - >TaPR1-44	specific	349400	29	164	3.26339e-89	256.792	CAP_PR-1
Q#67 - >TaPR1-67	specific	349400	29	164	7.76304e-88	253.325	CAP_PR-1
Q#68 - >TaPR1-68	specific	349400	29	164	6.37072e-89	256.021	CAP_PR-1
Q#69 - >TaPR1-69	specific	349400	29	164	6.87902e-89	256.021	CAP_PR-1
Q#70 - >TaPR1-70	superfamily	412178	26	147	2.10181e-28	102.327	CAP superfamily
Q#71 - >TaPR1-71	superfamily	412178	21	153	9.11561e-08	48.3989	CAP superfamily
Q#72 - >TaPR1-72	specific	349400	29	164	1.51068e-89	257.562	CAP_PR-1
Q#73 - >TaPR1-73	specific	349400	33	169	7.59193e-81	235.221	CAP_PR-1
Q#74 - >TaPR1-74	specific	349400	28	164	2.37339e-78	228.672	CAP_PR-1
Q#75 - >TaPR1-75	superfamily	412178	26	147	7.84188e-25	93.082	CAP superfamily
Q#76 - >TaPR1-76	specific	349400	29	164	1.60588e-90	259.873	CAP_PR-1
Q#77 - >TaPR1-77	specific	349400	28	168	1.58805e-78	229.443	CAP_PR-1
Q#78 - >TaPR1-78	specific	349400	28	168	2.8081e-78	228.672	CAP_PR-1
Q#79 - >TaPR1-79	specific	349400	28	168	1.58805e-78	229.443	CAP_PR-1
Q#80 - >TaPR1-80	specific	270968	428	693	1.36868e-87	276.846	STKc_IRAK
Q#80 - >TaPR1-80	specific	349400	30	166	8.73561e-77	243.31	CAP_PR-1
Q#80 - >TaPR1-80	specific	349400	180	315	5.28355e-73	233.295	CAP_PR-1
Q#81 - >TaPR1-81	superfamily	412178	50	185	1.15719e-56	174.744	CAP superfamily
Q#82 - >TaPR1-82	superfamily	412178	50	185	1.08745e-55	172.433	CAP superfamily
Q#83 - >TaPR1-83	superfamily	412178	48	183	2.11783e-56	173.974	CAP superfamily
