# Supplementary material for: Re-Examination Characterization and Screening of Stripe Rust Resistance Gene of Wheat TaPR1 Gene Family Based on the Transcriptome in Xinchun 32
Source: Int J Mol Sci. 2025 Jan 14;26(2):640. doi: 10.3390/ijms26020640 (PMC11766189; doi:10.3390/ijms26020640)
Supplement: Supplementary file 1 [file ijms-26-00640-s001.zip › Table S4.pdf]

Table S4. Statistical results of transcriptome sequencing data quality.

Sample	RawReads	RawBases	CleanReads	CleanBases	CleanRatio	Q20	Q30	GC
CK_0h1	69568210	10435231500	69541876	10371003052	99.96%	99.52%	98.12%	52.78%
CK_0h2	66769996	10015499400	66743712	9950620574	99.96%	99.47%	97.97%	53.84%
CK_0h3	74125522	11118828300	74096766	11032454854	99.96%	99.53%	98.19%	54.33%
C_120h1	70805460	10620819000	70779804	10567057492	99.96%	99.49%	98.03%	51.62%
C_120h2	74236532	11135479800	74209718	11071935336	99.96%	99.49%	98.02%	53.46%
C_120h3	66766384	10014957600	66743136	9945210064	99.97%	99.58%	98.33%	51.68%
C_240h1	69833916	10475087400	69809976	10410627956	99.97%	99.56%	98.21%	47.12%
C_240h2	68693680	10304052000	68671222	10228927692	99.97%	99.54%	98.14%	47.46%
C_240h3	77072466	11560869900	77045538	11494287460	99.97%	99.53%	98.13%	48.51%
C_24h1	67113832	10067074800	67090034	9989293102	99.96%	99.51%	98.08%	51.84%
C_24h2	72990082	10948512300	72960824	10876121390	99.96%	99.52%	98.14%	53.25%
C_24h3	70628154	10594223100	70602172	10522858008	99.96%	99.52%	98.12%	52.17%
C_360h1	64503350	9675502500	64480362	9628612030	99.96%	99.41%	97.67%	49.63%
C_360h2	73928406	11089260900	73902452	11026811126	99.96%	99.54%	98.15%	49.31%
C_360h3	76065374	11409806100	76039268	11318098380	99.97%	99.53%	98.16%	50.49%
C_48h1	75116496	11267474400	75090096	11202843260	99.96%	99.52%	98.14%	54.27%
C_48h2	65467568	9820135200	65443504	9775355030	99.96%	99.48%	98.01%	53.95%
C_48h3	64411440	9661716000	64387628	9617852744	99.96%	99.47%	97.97%	54.09%
