# Supplementary material for: Re-Examination Characterization and Screening of Stripe Rust Resistance Gene of Wheat TaPR1 Gene Family Based on the Transcriptome in Xinchun 32
Source: Int J Mol Sci. 2025 Jan 14;26(2):640. doi: 10.3390/ijms26020640 (PMC11766189; doi:10.3390/ijms26020640)
Supplement: Supplementary file 1 [file ijms-26-00640-s001.zip › Table S5.pdf]

Table S5. Screening and comparison of differential genes between different groups.

SampleID	Total	Up	Down
C1_vs_CK	19308	9054	10254
C2_vs_CK	9908	4445	5463
C3_vs_CK	12237	6024	6213
C4_vs_CK	19591	9832	9759
C5_vs_CK	19706	10872	8834
