# Supplementary material for: Re-Examination Characterization and Screening of Stripe Rust Resistance Gene of Wheat TaPR1 Gene Family Based on the Transcriptome in Xinchun 32
Source: Int J Mol Sci. 2025 Jan 14;26(2):640. doi: 10.3390/ijms26020640 (PMC11766189; doi:10.3390/ijms26020640)
Supplement: Supplementary file 1 [file ijms-26-00640-s001.zip › Table S6.pdf]

Table S6. Primers used in this experiment for qRT-PCR.

No.	Gene_ID	Primer (5'to3')	TM(℃)
1	26S-F	GCTGGCTCGTTCAACTGATG	60
	26S-R	GGACCAAGCGTTCTGATTACTC	
2	*TraesCS5B03G0483900*-F	ACTACCTCTCACCTCACA	64
	TraesCS5B03G0483900-R	GTCGTTGATCCTCTGGTT	
3	*TraesCS7B03G0276600*-F	GGCATCTTCCAAGAGTAGT	60
	TraesCS7B03G0276600-R	GCAGATTGACGAAGTCCT	
4	TraesCS7A03G0469500-F	CGAGAAGCAGAACTACCA	64
	TraesCS7A03G0469500-R	GTTGCAGGTGATGAAGAC	
5	TraesCS7D03G0450000-F	TTCGGCGAGAACATCTTC	62
	TraesCS7D03G0450000-R	TTGCTGTTGAGGTGGTAG	
6	TraesCS7D03G0362800-F	CGAAAAGAAGGACTACGACTA	64
	TraesCS7D03G0362800-R	AAGACGCCGAGGTTATTG	
7	TraesCS7D03G0450100-F	ACTACCATCTTGACAGCA	64
	TraesCS7D03G0450100-R	GTTGCAGGTGATGAAGAC	
8	TraesCS3A03G1118700-F	GAATATGCGGTTCTTGGACAT	60
	TraesCS3A03G1118700-R	CTCAGCCATCATTGTTTCAGA	
9	TraesCS5B03G1087600-F	CTGATACATACTCCCGATGG	64
	TraesCS5B03G1087600-R	GTACTGCTTCTCCGACAC	
10	TraesCS1D03G0729600-F	AAGGTGAAGGAGATCAAGAACGG	63
	TraesCS1D03G0729600-R	GAGGTGGTCGGCAAGGTT	
11	TraesCS5A03G0362200-F	CAAGTACCCAGTGTTCCA	64
	TraesCS5A03G0362200-R	CATTCTCTCCGACAACCT	
12	TraesCS3D03G0238500-F	GCCGACACGGAGAAGATC	64
	TraesCS3D03G0238500-R	TGGATGGTCTTGGGCTTC	
13	TraesCS6D03G0175200-F	ACTTGGTTGTGTCTTTCC	60
	TraesCS6D03G0175200-R	AGACCACCCTCACTAAAG	
14	TraesCS2B03G0306700-F	CACTTCCACGACTGCTTC	63
	TraesCS2B03G0306700-R	CTGCGTCTTGATGCTGTC	
15	TraesCS3B03G1319800-F	GTGCGTGACCAGAACAAC	64
	TraesCS3B03G1319800-R	TCTCGGAAATCACCACCTT	
16	TraesCS3D03G0029500-F	GTCTTCTTCAGGGTGTTCA	63
	TraesCS3D03G0029500-R	TCTTGGTCTTGGCTATGTC	
17	TraesCS2D03G1339300-F	ATGGACTTCTTCGGGAAC	64
	TraesCS2D03G1339300-R	CTGGATGTACTCGTCGTC	
