# Supplementary figures and images for: Re-Examination Characterization and Screening of Stripe Rust Resistance Gene of Wheat TaPR1 Gene Family Based on the Transcriptome in Xinchun 32
Source: Int J Mol Sci. 2025 Jan 14;26(2):640. doi: 10.3390/ijms26020640 (PMC11766189; doi:10.3390/ijms26020640)

**Figure S1. Analysis of cis-acting elements of *TaPR1* gene family.**

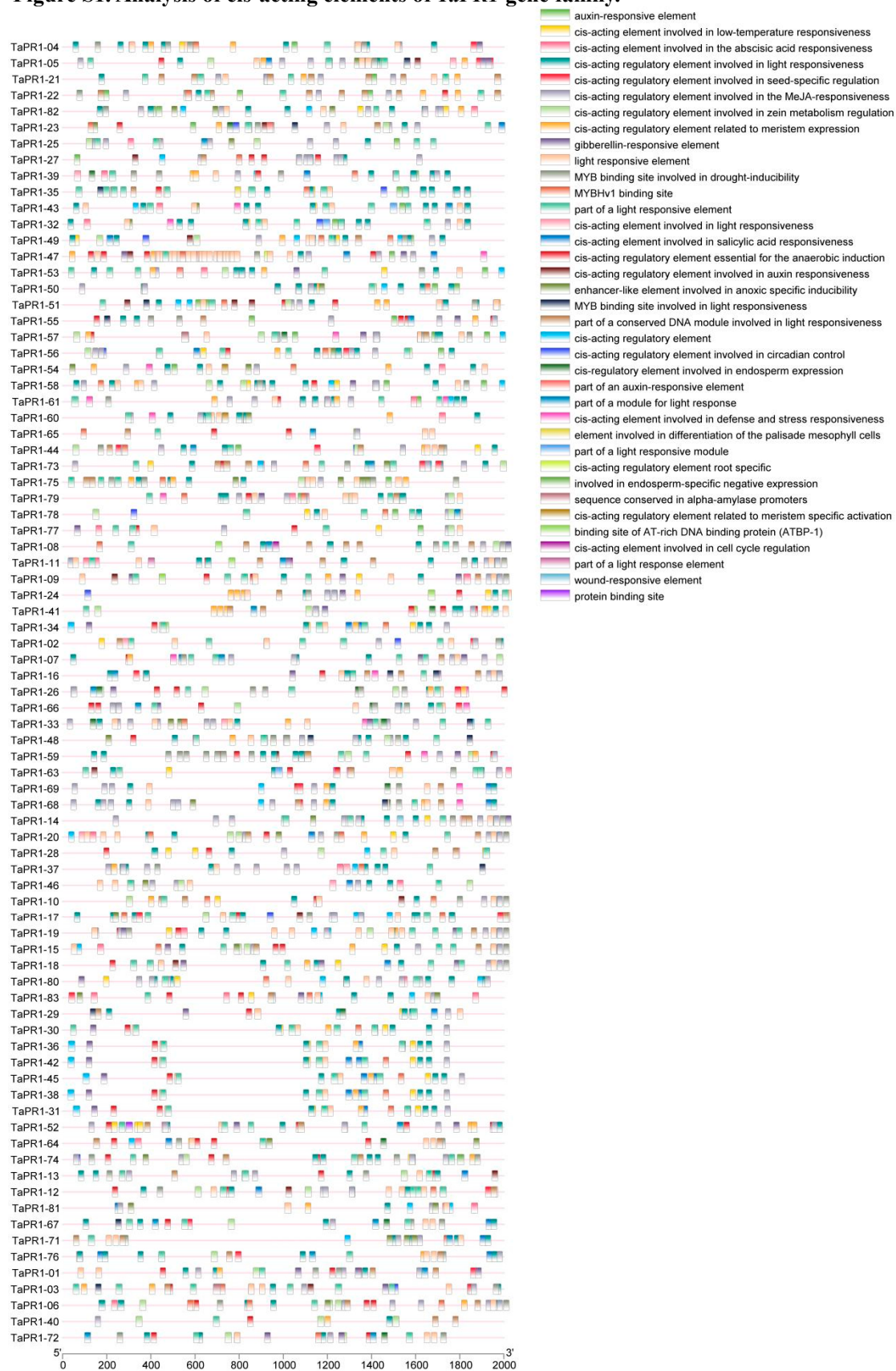

Supplement: Supplementary file 1 [file ijms-26-00640-s001.zip › Figure S1.pdf]
